# Facile Approach to Synthesize Gold Nanorod@Polyacrylic Acid/Calcium Phosphate Yolk–Shell Nanoparticles for Dual-Mode Imaging and pH/NIR-Responsive Drug Delivery

**DOI:** 10.1007/s40820-017-0155-3

**Published:** 2017-10-13

**Authors:** Guilan Li, Yidan Chen, Lingyu Zhang, Manjie Zhang, Shengnan Li, Lu Li, Tingting Wang, Chungang Wang

**Affiliations:** 1 Faculty of Chemistry, Northeast Normal University, Changchun, 130024 People’s Republic of China; 2 School of Chemistry and Environmental Engineering, Changchun University of Science and Technology, Changchun, 130022 People’s Republic of China

**Keywords:** Yolk–shell structure, Calcium phosphate, Dual-mode imaging, Photothermal therapy, Drug delivery

## Abstract

A facile strategy to fabricate gold nanorod@polyacrylic acid/calcium phosphate (AuNR@PAA/CaP) yolk–shell nanoparticles (NPs) composed with a PAA/CaP shell and an AuNR yolk is reported. The as-obtained AuNR@PAA/CaP yolk–shell NPs possess ultrahigh doxorubicin (DOX) loading capability (1 mg DOX/mg NPs), superior photothermal conversion property (26%) and pH/near-infrared (NIR) dual-responsive drug delivery performance. The released DOX continuously increased due to the damage of the CaP shell at low pH values. When the DOX-loaded AuNR@PAA/CaP yolk–shell NPs were exposed to NIR irradiation, a burst-like drug release occurs owing to the heat produced by the AuNRs. Furthermore, AuNR@PAA/CaP yolk–shell NPs are successfully employed for synergic dual-mode X-ray computed tomography/photoacoustic imaging and chemo-photothermal cancer therapy. Therefore, this work brings new insights for the synthesis of multifunctional nanomaterials and extends theranostic applications.

**Electronic supplementary material:**

The online version of this article (doi:10.1007/s40820-017-0155-3) contains supplementary material, which is available to authorized users.

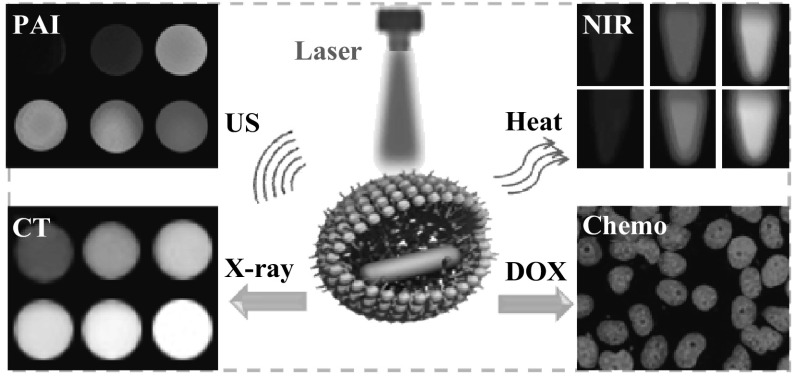

## Highlights

- We report a facile strategy to fabricate gold nanorod@polyacrylic acid/calcium phosphate (AuNR@PAA/CaP) yolk–shell nanoparticles.
- The as-obtained AuNR@PAA/CaP yolk–shell nanoparticles (NPs) possess ultrahigh doxorubicin (DOX) loading capability (1 mg DOX/mg NPs), superior photothermal conversion property (26%) and pH/near-infrared (NIR) dual-responsive drug delivery performance, which were employed for synergic dual-mode X-ray computed tomography (CT)/photoacoustic (PA) imaging and chemo-photothermal cancer therapy.
- This work brings new insights for the synthesis of multifunctional nanomaterials and extends theranostic applications.

## Introduction

Multifunctional nanoparticles (NPs) with complementary capacities of multimodal imaging and therapeutic functions have drawn extensive attention in biomedical areas [1–6]. Nowadays, calcium phosphate (CaP) NPs have gained increasing attention in anticancer drug delivery because of their excellent biocompatibility and pH-sensitivity, originating from their chemical nature and mimics the inorganic component of biological hard tissues, such as bone and tooth [7–11]. However, owing to the lack of the theranostic capability, single CaP NPs are difficult to achieve simultaneous imaging and cancer theranostics. The main strategy turns to synthesize CaP-based multifunctional NPs with the capability of diagnosis and therapeutics. For example, Liu et al. developed a synthetic route to obtain amphiphilic gelatin–iron oxide core/CaP shell NPs, integrating magnetic resonance imaging and chemotherapy for killing cancer cells [12]. It should be noted that sole modality imaging or therapy cannot enhance the anticancer efficiency in comparison with multimodal imaging and multiple therapeutic. Therefore, it is absolutely imperative to propose a simple synthetic method for fabricating CaP-based multifunctional NPs that possess the capacities of simultaneous dual-mode imaging diagnosis and chemo-photothermal therapy.

Among numerous photothermal nanomaterials, the optical property of gold nanorod (AuNRs) presented good photothermal therapy (PTT) effect owing to their tunable localized surface plasmon resonance (LSPR) across the NIR region [13–27]. Meanwhile, AuNRs possess photoacoustic (PA) and X-ray computed tomography (CT) imaging capacity, due to the strong near-infrared (NIR) absorption and X-ray opacity [28, 29]. Recently, Lu and co-workers prepared yolk–shell AuNR@hollow periodic mesoporous organosilica nanospheres only for chemo-photothermal therapy of breast cancer [30]. However, their materials still have problems that need to be tackled including large particle size, poor biocompatibility and low drug loading capability. Moreover, up to now, there have been no reports on the synthesis of CaP-based yolk–shell NPs composed of a CaP shell and a removable AuNR yolk. It is worth mentioning that the yolk–shell architecture is a promising candidate for developing drug loading systems compared with the core–shell structure, owing to the unique cavity, large surface area and excellent loading capacity [31–41]. Hence, developing a facile synthetic strategy to fabricate well-dispersed AuNR core/CaP shell NPs with ultrahigh doxorubicin (DOX) loading capability and pH/NIR dual-responsive drug delivery performance for dual-mode CT/PA imaging and synergic chemo-photothermal therapy of cancer cells still remains a great challenge.

Herein, we have successfully developed for the first time a mild and facile route to fabricate AuNR@PAA/CaP yolk–shell NPs for synergistic dual-mode CT/PA imaging and chemo-photothermal therapy of cancer cells.

## Experimental Details

### Materials

Hydrogen tetrachloroaurate trihydrate (HAuCl_4_·3H_2_O), silver nitrate (AgNO_3_), hexadecyltrimethylammonium bromide (CTAB), L-ascorbic acid (LAA), sodium borohydride (NaBH_4_), polyacrylic acid (PAA, *M*_W_ ≈ 1800) and doxorubicin hydrochloride (DOX) were purchased from Sigma-Aldrich (USA). Disodium hydrogen phosphate (Na_2_HPO_4_), hydrochloric acid (HCl, 37% weight in water), tetraethylorthosilicate (TEOS), methanol (HCHO, 37% weight in water), anhydrous ethanol (CH_3_CH_2_OH), sodium hydroxide (NaOH), calcium hydroxide (Ca(OH)_2_) and isopropyl alcohol (IPA) were purchased from Sinopharm Chemical Reagent Beijing Co. Ltd. All chemicals were used without any further purification. All glassware was first cleaned with freshly prepared aqua regia and extensively rinsed with water before it was used. The deionized (DI) water was used in all experiments.

### Characterization

Transmission electron microscope (TEM) measurements were taken on a JEOL-2100F transmission electron microscope at 200 kV (Hitachi, Japan). The irradiation was performed using a NIR laser with a center wavelength of 808 nm (Beijing Kaipulin Optoelectronic Technology Co.). The scanning electron microscopy (SEM) and energy dispersive X-ray (EDX) spectrum were carried out with a JEOL JSM-7610F scanning electron microscope. X-ray photoelectron spectra (XPS) were measured on an ECSALAB 250 using non-mono-chromatized Al-*K*α radiation. Fourier transform infrared (FTIR) spectra were performed by a Magna 560 FTIR spectrometer (Nicolet, USA). The UV–Vis spectra were recorded at room temperature on a Japan JASCO V-570 spectrometer fluorescence spectrophotometer. The inductively coupled plasma atomic emission spectroscopy (ICP-AES) was determined by a Leeman ICP-AES Prodigy instrument.

### Synthesis of AuNRs

AuNRs with an aspect ratio of ~4 was prepared by a seed-mediated growth method using CTAB surfactants as reported previously [42]. Firstly, the synthesis of gold nanoseeds: HAuCl_4_ (10 mM, 0.25 mL) and CTAB (0.1 M, 10 mL) were added into a 10 mL glass bottle with gentle mixing before a freshly prepared and ice-bathed NaBH_4_ solution (10 mM, 0.6 mL) was injected and then the mixture was magnetically stirred until the color changed from golden yellow to brown indicating that 3.5 nm gold nanoseeds were obtained. The seed solution was then kept at 30 °C for at least 2 h before usage. Secondly, the growth of gold nanorods: AgNO_3_ (10 mM, 1 mL) and HAuCl_4_ (10 mM, 5 mL) were added into CTAB (0.1 M, 100 mL) in a 250 mL glass bottle, followed by the addition of HCl (0.1 M, 700 μL), LAA (0.1 M, 700 μL) and gold seeds (100 μL). The growth solution was left at 30 °C overnight. Finally, CTAB-stabilized AuNRs were prepared, then the sample solution was centrifuged several times to remove superfluous CTAB and redispersed into 100 mL of DI water for further use.

### Synthesis of AuNR@mSiO_2_ Core–Shell NPs

When the pH value of 16 mL AuNRs solution was adjusted to about 10 with NaOH (0.1 M) solution under stirring, 30 μL of TEOS was subsequently injected slowly under vigorous stirring. The reaction mixture was allowed to proceed for 4 h to form an approximately 15-nm thick silica layer on the surface of AuNRs. Finally the AuNR@mSiO_2_ core–shell NPs were isolated by centrifugation and washed with DI water several times and then re-dissolved in 5 mL DI water for further use.

### Synthesis of AuNR@PAA/CaP Yolk–Shell NPs

In a 50 mL of flask, 8 mg Ca(OH)_2_ and 100 μL of PAA aqueous solution (0.2 g mL^−1^) were firstly added to 10 mL DI water under magnetic stirring. In succession, 5 mL of AuNR@mSiO_2_ core–shell NPs solution was dispersed into the solution to form a suspension. Then, 20 mL of IPA was dripped into the suspension under magnetic stirring. Afterward, 24 mg Na_2_HPO_4_ was added to the above mixed suspension under magnetic stirring for 10 h. After being centrifuged and washed with DI water, the AuNR@mSiO_2_@PAA/CaP core–shell NPs were obtained. Finally, after etching the mSiO_2_ layer, the AuNR@PAA/CaP yolk–shell NPs were obtained.

### DOX Loading and Release

The amount of DOX loaded into the AuNR@PAA/CaP yolk–shell NPs was measured by UV–Vis spectrophotometer. After mixing DOX (0.1 mL, 10 mg mL^−1^) with AuNR@PAA/CaP yolk–shell NPs (0.8 mL, 1.2 mg mL^−1^), and shaking overnight, DOX-loaded AuNR@PAA/CaP yolk–shell NPs were obtained by centrifugation and washed three times with DI water to remove the DOX adsorbed on the surface. Free DOX in the supernatant was determined by measuring the absorbance at 480 nm in a UV–Vis spectrophotometer. The DOX-loading efficiency (LE) was calculated by Eq. 1:

1 $${\text{LE}}(\% ) = \frac{{W_{{{\text{initial}}\,\,{\text{DOX}}}} \, - \,W_{{{\text{remanent}}\,\,{\text{DOX}}}} }}{{W_{\text{AuNR@PAA/CaP}} }} \times 100\% \,\,\,\,\,\text{ }({\text{mg}}\,{\text{mg}}^{ - 1} )$$

Two portions of the prepared DOX-loaded AuNR@PAA/CaP yolk–shell NPs at equal amount were redispersed in pH 7.4 and pH 5.0 PBS (0.5 mL) and then transferred into pretreated semipermeable dialysis bags at 37 °C, respectively. After the two bags were immersed into 5 mL of PBS buffer (pH 7.4 and pH 5.0) at 37 °C, the amount of released DOX moving into the solution was determined by measuring the absorbance at 480 nm in a UV–Vis spectrophotometer at selected time intervals. To confirm that the laser irradiation can induce the drug release, another experiment was also carried out under the same procedures. The sample was immersed in PBS buffer at pH 5.0 with NIR irradiation (808 nm, 1.0 W cm^−2^) at selected time intervals. DOX concentration in the supernatant was determined by UV–Vis spectrophotometer as well. The samples (1 mg) were put into pH 5.0 PBS (3 mL). The supernatants were collected by centrifugation at selected time intervals and analyzed by ICP-AES to measure the Ca content.

### Cell Culture

Human hepatocellular carcinoma (HeLa) cells were grown as a monolayer in a humidified incubator at 37 °C in a 95% air 5% CO_2_ in Dulbecco’s modified eagle medium (DMEM) supplemented with 10% fetal bovine serum.

### The Photothermal Therapy of AuNR@PAA/CaP Yolk–Shell NPs

The photothermal effect of AuNR@PAA/CaP yolk–shell NPs was measured in aqueous solution. Briefly, AuNR@PAA/CaP yolk–shell NPs (1 mL) with various concentrations were exposed to the 808-nm NIR laser (power density 1.0 W cm^−2^) for 5 min. The temperature was recorded every 30 s. Subsequently, the photothermal effect in the cell level was analyzed by using calcein AM staining method. Calcein AM can only penetrate in live cells and emit green fluorescence. The cells were seeded in a 24-well plate (2.5 × 10^4^ cells per well) for 24 h. Then, the cells were divided into four groups: group 1 with PBS only; group 2 incubated with AuNR@PAA/CaP yolk–shell NPs (25 μg mL^−1^); group 3 incubated with NIR; group 4 incubated with both NPs and NIR laser. The NIR laser used in this experiment was 1.0 W cm^−2^ and the irradiation time was 5 min. After all the treatment, the cells were finally stained with calcein AM.

### Calculation of the Photothermal Conversion Efficiency (*η*)

To evaluate the photothermal conversion efficiency (*η*), the time-dependent temperature increment of the aqueous dispersion (0.5 mM) was recorded under the continuous 808-nm NIR laser irradiation with a power density of 1.0 W cm^−2^. Subsequently, the irradiation source was shut off, and the temperature decrease of the aqueous dispersion was monitored to determine the rate of heat transfer from the dispersion system to the environment. The photothermal conversion efficiency (*η*) of AuNR@PAA/CaP yolk–shell NPs was calculated using Eq. 2:

2 $$\eta \, = \,\frac{{hS(T_{\hbox{max} } \, - \,T_{\text{surr}} )\, - \,Q_{\text{dis}} }}{{I(1 - 10^{{ - A_{808} }} )}}$$

where *h* is the heat transfer coefficient, *S* is the surface area of the container, *T*_max_ and *T*_surr_ are the equilibrium temperature and ambient temperature, respectively. *Q*_dis_ represents the heat dissipation from the light absorbed by container, *I* is the incident laser power, and *A*_808_ is the absorption intensity of AuNR@PAA/CaP yolk–shell NPs at 808 nm. The value of *hS* is derived according to Eq. 3:

3 $$\tau_{s} = \frac{{m_{\text{D}} C_{\text{D}} }}{hs}$$

where *τ*_s_ is the sample system time constant, *m*_D_ and *C*_D_ are the mass and heat capacity of DI water used as the solvent, respectively. The *Q*_dis_ was measured independently using sample cell containing pure water without AuNR@PAA/CaP yolk–shell NPs.

### In Vitro Cytotoxicity Evaluation Against HeLa Cells

The cytotoxicity of empty AuNR@PAA/CaP yolk–shell NPs, free DOX and DOX-loaded AuNR@PAA/CaP yolk–shell NPs were evaluated by standard 3-(4,5-dimethylthialzol-2-yl)-2,5-diphenyltetrazolium bromide (MTT) assays. HeLa cells were seeded in a 96-well plate at a density of 2.5 × 10^4^ per well and incubated in the atmosphere of 95% air and 5% CO_2_ at 37 °C with 10% fetal bovine serum for 24 h. Then various concentrations of empty AuNR@PAA/CaP yolk–shell NPs, free DOX and DOX-loaded AuNR@PAA/CaP yolk–shell NPs were added, respectively. In another plate, the suspensions of AuNR@PAA/CaP yolk–shell NPs with different concentrations were added into HeLa cells with the 808-nm NIR laser irradiation for 5 min. One row of a 96-well plate was added culture medium only to be a blank control. After incubated for 24 h, 20 μL of 5 mg mL^−1^ MTT solution was added to each well for 4 h incubation. Then, the medium was replaced with DMSO (150 μL) to dissolve the MTT formazan crystals. The cell viability can be calculated by using Eq. 4:

4 $${\text{Cell viability}}(\% )\, = \,\frac{{A_{{({\text{test}}\,\,{\text{cells}})}} }}{{A_{{({\text{control}}\,\,{\text{cells}})}} }}\, \times \,100\% $$

### CT Imaging of AuNR@PAA/CaP Yolk–Shell NPs In Vitro

AuNR@PAA/CaP yolk–shell NPs with the Au concentrations in a range of 0–0.25 M were poured into tubes and placed in a self-made scanning holder. CT images were acquired under the following parameters: thickness, 1.0 mm; pitch, 120 kV, 280 mA; field of view, 300 mm; gantry rotation time, 4.95 s. CT data were analyzed by recording the Hounsfield units (HUs) for regions of interest. The raw data were reconstructed using 3D-Med software to acquire the CT images and calculate the CT values.

### PA Signal Measurement In Vitro

To test the linearity of the PA signal as a function of AuNR@PAA/CaP yolk–shell NPs, 0.2 mL of the AuNR@PAA/CaP yolk–shell NPs aqueous suspension with different Au concentrations (0, 0.4, 0.5, 0.7 and 0.9 mM) was added to the agar-phantom container and placed in the MOST inVision 128 (iThera) system for signal detection. A complete PA image of the phantom was collected at 808 nm.

## Results and Discussion

### Synthesis and Characterization of AuNR@PAA/CaP Yolk–Shell NPs

**Scheme 1:**
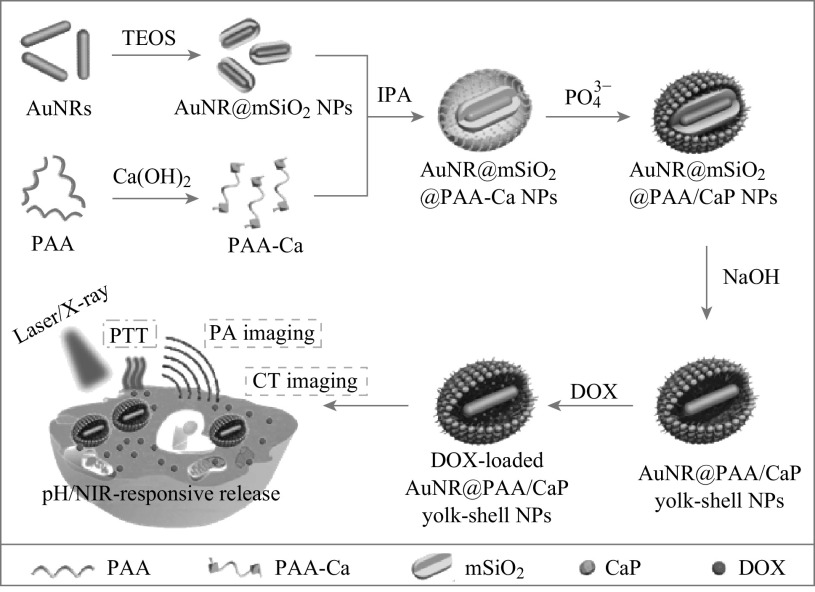
Schematic illustration of the synthetic strategy for the AuNR@PAA/CaP yolk–shell NPs as pH/NIR-responsive drug carriers for simultaneous dual-mode CT/PA imaging and chemo-photothermal therapy in vitro

As illustrated in Scheme 1, AuNR@mesoporous silica (AuNR@mSiO_2_) core–shell NPs were formed upon the addition of TEOS into the prepared AuNR solution. The as-fabricated AuNR@mSiO_2_ core–shell NPs mixed with the PAA-Ca aqueous solution, which obtained by adding Ca(OH)_2_ powder into PAA aqueous solution. Upon the addition of IPA, the PAA-Ca self-assembled around the surface of AuNR@mSiO_2_ core–shell NPs to obtain AuNR@mSiO_2_@PAA-Ca core–shell NPs. Then, AuNR@mSiO_2_@PAA/CaP core–shell NPs were fabricated through the assistance of Na_2_HPO_4_ as the phosphate anion source. Finally, the mSiO_2_ layer was etched by sodium hydroxide from the core–shell AuNR@mSiO_2_@PAA/CaP NPs to get the yolk–shell AuNR@PAA/CaP NPs. After loading the anticancer drug, DOX, the DOX-loaded yolk–shell NPs were utilized as pH/NIR-responsive drug vehicles for dual-mode CT/PA imaging and chemo-photothermal cancer therapy in vitro.

**Fig. 1:**
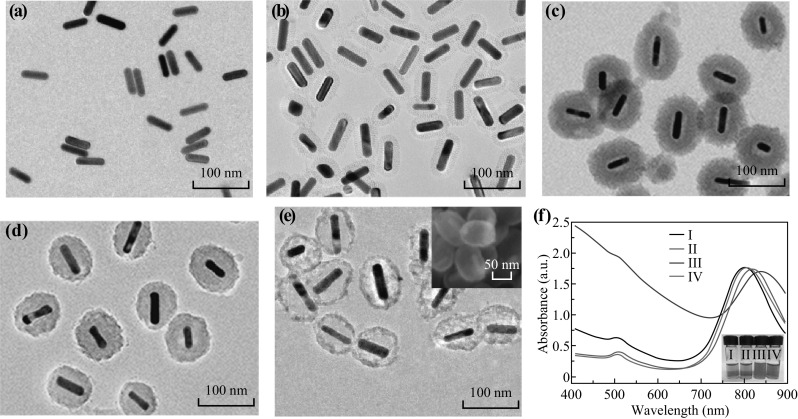
TEM images of **a** AuNRs, **b** AuNR@mSiO_2_ core–shell NPs, **c** AuNR@mSiO_2_@PAA-Ca core–shell NPs, **d** AuNR@mSiO_2_@PAA/CaP core–shell NPs, **e** AuNR@PAA/CaP yolk–shell NPs, the inset shows the SEM image of the yolk–shell NPs, **f** UV–Vis absorbance spectra of (I) AuNRs, (II) AuNR@mSiO_2_ core–shell NPs, (III) AuNR@mSiO_2_@PAA/CaP core–shell NPs and (IV) AuNR@PAA/CaP yolk–shell NPs

The TEM image in Fig. 1a shows that the average length and width of AuNRs are around 60 and 15 nm, respectively (about 4:1 aspect ratio). The absorption wavelength of AuNRs can be tuned by changing the AuNR aspect ratio [43]. When the aspect ratio is around 4, a strong LSPR band was observed at around 800 nm, matching the 808-nm NIR laser irradiation well, thereby ensuring effective heat production. With the addition of TEOS and ammonium hydroxide into the as-prepared AuNR solution, the AuNR@mSiO_2_ core–shell NPs are about 80 nm in length and 45 nm in width (Fig. 1b). To synthesize monodispersed AuNR@mSiO_2_@PAA-Ca core–shell NPs, the PAA-Ca aqueous solution was firstly obtained by adding Ca(OH)_2_ powder into PAA aqueous solution due to the acid–base neutralization reaction [2]. Then, the as-fabricated AuNR@mSiO_2_ core–shell NPs and IPA were added to the PAA-Ca aqueous solution in sequence under vigorous stirring to form AuNR@mSiO_2_@PAA-Ca core–shell NPs with a PAA-Ca shell about 10 nm (Fig. 1c), due to the insolubility of PAA-Ca in the presence of IPA. The subsequent addition of Na_2_HPO_4_ powder as the phosphate anion source induced the formation of a PAA-CaP shell, the finally gained well-dispersed AuNR@mSiO_2_@PAA/CaP core–shell NPs with the average length and width around 100 and 65 nm, respectively (Fig. 1d). Then, the resulting AuNR@mSiO_2_@PAA/CaP core–shell NPs were treated in diluted base solution for 1 h to generate AuNR@PAA/CaP yolk–shell NPs with a thin PAA/CaP shell of approximately 10 nm in thickness (Fig. 1e), which is shown in Fig. 1c. The oval-shaped cavity formation with the lower contrast in the TEM image made the hollow structure pronounced and proved the synthesis of yolk–shell NPs. The SEM image of the AuNR@PAA/CaP yolk–shell NPs presents a fairly rough surface, which proved the shell is composed of a large amount of small CaP. The formation process of the AuNR@PAA/CaP yolk–shell NPs was monitored by the UV–Vis absorbance spectra in Fig. 1f. The absorption peak around 510 nm is ascribed to the transverse surface plasmon band of the AuNRs, and the absorption peak around 802 nm is attributed to the LSPR band. Compared to the absorption spectrum of AuNRs, the LSPR bands of AuNR@mSiO_2_NPs and AuNR@mSiO_2_@PAA/CaP NPs redshift about 18 nm (from 802 to 820 nm) and 38 nm (from 802 to 840 nm), respectively, owing to the difference of the local dielectric function of surrounding medium. While, after dissolving mSiO_2_ layer, a strong LSPR band of AuNR@PAA/CaP yolk–shell NPs at 806 nm was observed. The above results further verify the successful preparation of AuNR@PAA/CaP yolk–shell NPs. In addition, no obvious aggregation was observed when the AuNR@PAA/CaP yolk–shell NPs were dispersed into water, PBS buffer and Dulbecco’s modified eagle medium (DMEM) for 4 h, respectively, reflecting their good stability (Fig. S1, ESI).

To further confirm the details of the AuNR@PAA/CaP yolk–shell NPs, a series of characterization were performed. Figure S2 displays the FTIR spectrum of the AuNR@PAA/CaP yolk–shell NPs. The broad peak around 3500 cm^−1^ originates from moisture in the samples. The absorption bands at 1558 and 1419 cm^−1^ are attributed to the characteristic peaks of the carbonyl (C = O) of the carboxylic acid (–COO–) group, confirming the presence of PAA. Moreover, the characteristic peaks appeared at 565 and 1087 cm^−1^ can be assigned to the characteristic peaks of O–P–O bending and asymmetric stretching of PO_4_^3−^ ions. To further verify the crystallinity of obtained AuNR@PAA/CaP yolk–shell NPs, the XRD pattern of the sample was carried out. The diffraction peaks well match the planes of Au crystals, indicating the formation of crystalline Au. In addition, the characteristic peak at around 25°–35° is assigned to the amorphous CaP (Fig. S3, ESI). Surface information of the AuNR@PAA/CaP yolk–shell NPs was characterized by XPS in Fig. S4. The peaks at 190 and 132 eV are assigned to P 2*s* and P 2*p*, respectively. The Ca 2*s* and Ca 2*p* peaks appear at binding energies of 438 and 347 eV, and the binding energies of 531 and 87 eV are attributed to O 1*s* and Au 4*f*, respectively. EDX spectrum confirms that the AuNR@PAA/CaP yolk–shell NPs are composed of Au, Ca, C, P, O (Fig. S5, ESI). Meanwhile, an ICP-AES analysis quantified the weight percentage of Au and Ca is 13.5% and 19.4%, respectively.

### Photothermal Effect

**Fig. 2:**
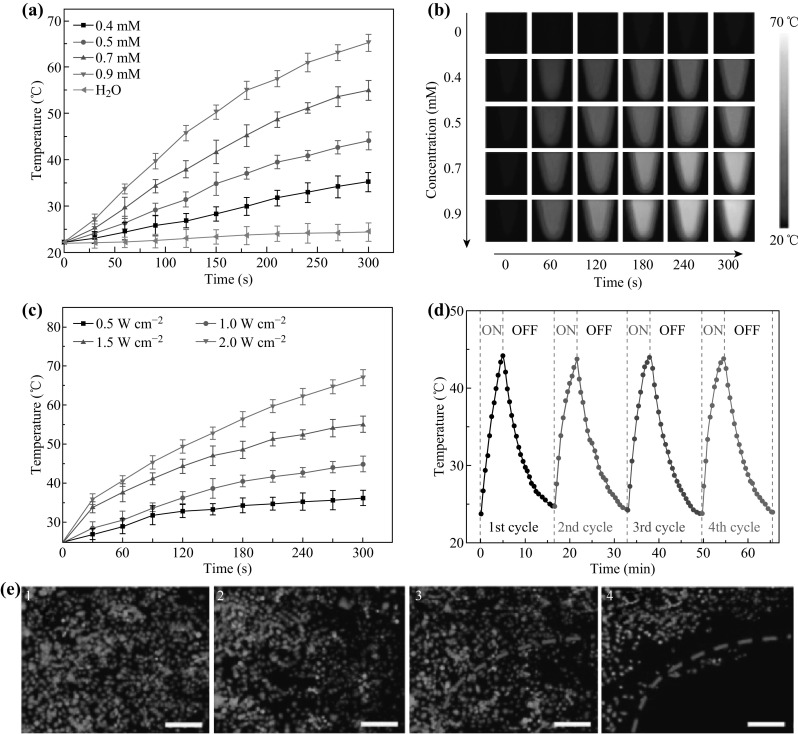
**a** Variation of temperature as a function of different Au concentrations irradiated with an 808-nm laser (power density: 1.0 W cm^−2^), **b** Photothermal images of the yolk–shell NPs solution at different Au concentrations, and pure water exposed to the 808-nm laser (1.0 W cm^−2^) recorded at different time intervals, **c** NIR-induced temperature increase at various lasers power densities in aqueous solution (808 nm, 0.5 mM), **d** Temperature monitoring of the yolk–shell NPs aqueous suspension (the Au concentration: 0.5 mM) during for successive cycles of an on-and-off laser, **e** Fluorescence microscopy images of HeLa cells with different treatments via staining with calcein AM: (1) control; (2) yolk–shell NPs only; (3) laser irradiation only; (4) yolk–shell NPs with laser irradiation (scale bars: 200 μm)

The AuNR@PAA/CaP yolk–shell NPs display a strong absorption peak at 806 nm (Fig. 1f), making it possible to be a photothermal treatment agent. To acquaint the photothermal performance, the yolk–shell NPs with different Au concentrations (0, 0.4, 0.5, 0.7, and 0.9 mM at 1.0 W cm^−2^) were irradiated with a NIR laser (808 nm) for 300 s, and the temperature was monitored by a digital thermometer (Fig. 2a, b). Meanwhile, the concentration of 0.5 mM Au irradiated with various lasers power densities (0.5, 1.0, 1.5, and 2.0 W cm^−2^) was also performed in Fig. 2c. It is obviously found that the temperature increasing depends on the concentration, lasers power density and irradiation duration. The Au concentrations of solutions greater than 0.5 mM can be easily heated from 22 °C at least to 42 °C. It is known that cancer cells can be effectively killed above 42 °C. As a negative control group, the temperature of water is stable at 22 °C. In addition, the photothermal conversion efficiency (*η*) of the AuNR@PAA/CaP yolk–shell NPs was determined to be 26% (Fig. S6, ESI). As shown in Fig. 2d, no significant decrease of the temperature elevation is observed of the AuNR@PAA/CaP yolk–shell NPs after four repeated laser on-and-off cycles (the laser was on for about 5 min in each cycle) and the TEM image also indicates that the “rod-like” structure of AuNRs stay unchanged in the PAA/CaP shell (Fig. S7, ESI). Thus, our AuNR@PAA/CaP yolk–shell NPs possess good photostability and constant photothermal conversion behavior.

Next, to investigate the photothermal effect in vitro, the fluorescence microscopy images were obtained by staining the live cells with calcein AM, which can emit strong green fluorescence in live cells. As shown in Fig. 2e, when the HeLa cells were treated with both 808-nm laser irradiation and AuNR@PAA/CaP yolk–shell NPs, the dark region matches the laser irradiation area very well, suggesting the death of HeLa cells upon exposure to the laser irradiation. As a contrast, the cell viability and cell density are not reduced when the samples were treated by only the yolk–shell NPs or laser irradiation, compared with the control group. Obviously, the results illustrate that the yolk–shell NPs can transform laser energy into heat energy, which could kill HeLa cells and reduce adverse side effects to normal tissues as photothermal agents.

### DOX Loading, pH/NIR-Responsive Controlled Release and Cytotoxicity Assays in vitro

**Fig. 3:**
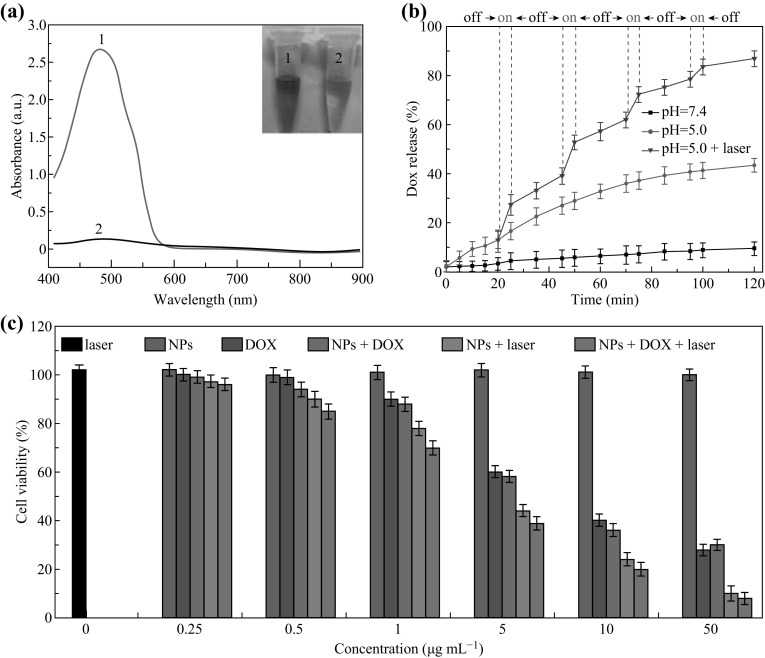
**a** UV–Vis absorption spectra and photographs (inset) of original DOX aqueous solution (1) and the residual DOX content in the supernatant after interaction with AuNR@PAA/CaP yolk–shell NPs (2); **b** DOX-release profiles of DOX from DOX-loaded yolk–shell NPs in PBS buffer: pH = 7.4, 5.0 and 5.0, with periodic laser ON/OFF irradiation (808 nm, 1.0 W cm^−2^), **c** Cytotoxicity assays of HeLa cells incubated with different treatments (laser, NPs, DOX, NPs + DOX, NPs + laser and NPs + DOX + laser, laser irradiation: 1.0 W cm^−2^, 5 min)

To further evaluate the drug loading capacity, pH/NIR controlled release behavior and cytotoxicity assays of the AuNR@PAA/CaP yolk–shell NPs, we selected DOX as a modal anticancer drug. The loading capacity of the AuNR@PAA/CaP yolk–shell NPs is evaluated by measuring the DOX concentration in solution before and after loading into the yolk–shell NPs using UV–Vis spectrophotometer measurements at 480 nm (Fig. 3a). The absorption intensity of DOX decreases significantly, indicating that the DOX has been stored in the yolk–shell NPs. The loading efficiency of DOX molecules into the AuNR@PAA/CaP yolk–shell NPs can reach up to approximately 100%, and the loading content is 1 mg of DOX per mg of the yolk–shell NPs. This ultrahigh loading capacity is major attributed to the void spaces, because of the thin CaP shell of about 10 nm, which can load a small amount of the drug molecules. Figure 3b shows different cumulative DOX-release profiles from the DOX-loaded AuNR@PAA/CaP yolk–shell NPs under different conditions at 37 °C. In neutral phosphate buffered saline (PBS), which is similar to the normal tissue environment (pH 7.4), only 10% of DOX is released within 2 h and there is almost no release afterward (Fig. S8, ESI). Whereas the cumulative drug release amount attains 45.8% within the same period at mildly acidic environment (pH 5.0), which is similar to the extracellular pH of tumors, owing to the dissolution of CaP in acid environment. In order to further verify the dissolution of CaP NPs, we measured the content of Ca in the PBS (pH 5.0 and pH 7.4) at different time points with ICP-AES (Fig. S9, ESI). The result shows that the content of Ca increases significantly with the extension of time (pH 5.0), confirming that the pH-responsive drug release of DOX results from the dissolution of CaP, while the extremely slow release of Ca occurs in pH 7.4. The released Ca ions will not make much difference on cells, and owing to the yolk–shell NPs’ high performance in photothermal effect and drug loading capacity, cancer cells can be ablated based on a small amount of DOX-loaded yolk–shell NPs (50 μg mL^−1^). Even the whole CaP shell is dissolved, and the Ca ions concentration is just ~1.7 × 10^−7^ M, which can still maintain a normal level [44]. In addition, we also investigated the promotional effect of NIR irradiation on DOX release during the experiment. In a certain time interval, the DOX-loaded yolk–shell NPs were exposed to an 808 nm NIR laser (pH 5.0). As shown in Fig. 3b, a burst release of DOX occurs upon applying the NIR laser. As a result of the photothermal effect of the yolk–shell NPs, the drug release upon NIR irradiation enhances significantly because the generated heat can effectively induce DOX release.

All the results demonstrate that the AuNR@PAA/CaP yolk–shell NPs can achieve pH/NIR dual-responsive release, making it possible for minimizing the drug stimulation to normal cells and improving antitumor efficacy. As depicted in Fig. 3c, the cell viability was as high as 98.2% when the HeLa cells were treated with AuNR@PAA/CaP yolk–shell NPs at a high concentration of 50 μg mL^−1^, proving that the yolk–shell NPs exhibit excellent biocompatibility. DOX-loaded AuNR@PAA/CaP yolk–shell NPs reveal a similar cell toxicity against HeLa cells with the same concentration of DOX, confirming that the yolk–shell NPs are effective drug vehicles. It should be noted that single yolk–shell NPs and DOX-loaded yolk–shell NPs under 808-nm laser irradiation, respectively, exhibit different ability for photothermal ablation of cancer cells, and the latter shows a higher cell toxicity. The results indicate that the combination of the chemo- and photothermal therapy of DOX-loaded AuNR@PAA/CaP yolk–shell NPs could significantly enhance the efficiency of tumor ablation.

### Confocal Laser Scanning Microscopy

**Fig. 4:**
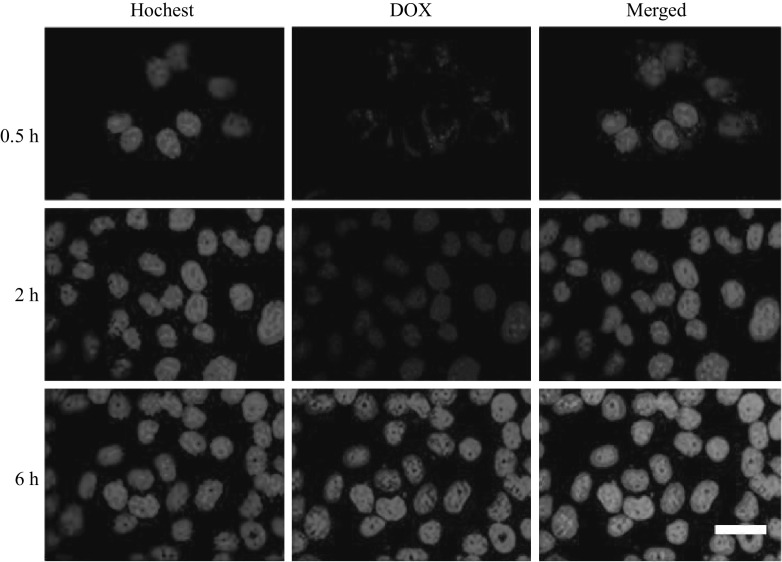
CLSM images of HeLa cells incubated with DOX-loaded AuNR@PAA/CaP yolk–shell NPs for 0.5, 2, and 6 h (scale bar: 10 μm)

To further examine the time-dependent cell uptake and the intracellular release behavior of DOX-loaded AuNR@PAA/CaP yolk–shell NPs, the confocal laser scanning microscopy (CLSM) images of HeLa cells incubated with DOX-loaded AuNR@PAA/CaP yolk–shell NPs at different time points (0.5, 2, and 6 h) were collected. As shown in Fig. 4, the CLSM images were obtained for the red fluorescence of DOX and the blue fluorescence of Hoechst 33,342 after the nuclei was stained. With the increase in the incubation time, the DOX molecules gathered in the cell nucleus gradually increased, which suggests that DOX-loaded NPs could facilitate cellular internalization and final delivery of DOX to the cell nuclei. We could conclude that the AuNR@PAA/CaP yolk–shell NPs have great potential as promising drug delivery nanocarriers to cancer cells.

### CT and PA Imaging in vitro

**Fig. 5:**
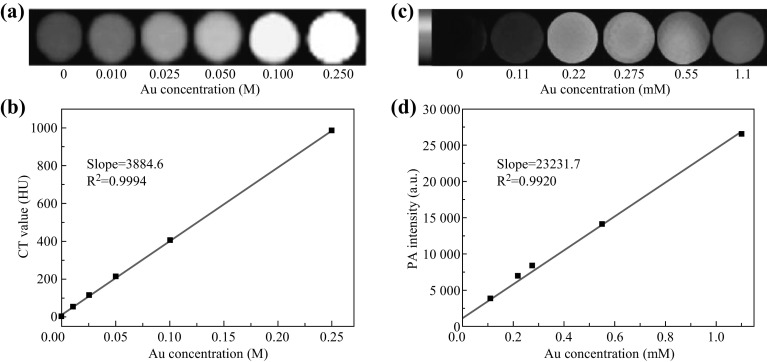
**a** CT images of AuNR@PAA/CaP yolk–shell NPs and **b** linear relationship of CT signal intensities versus Au concentrations, **c** PA images and **d** linear relationship of PA signal intensities with different Au concentrations

The Au-based NPs have been considered as a promising CT imaging contrast agent, due to the presence of Au which has a higher X-ray absorption coefficient than iodine agents of conventional CT [45–47]. Therefore, we assessed the CT contrast efficacy of the AuNR@PAA/CaP yolk–shell NPs. The CT signals enhance with increasing NPs concentrations (Fig. 5a). The HUs exhibit a well-correlated linear relationship in Fig. 5b (*R* ^2^ = 0.9994). These results suggest that AuNR@PAA/CaP yolk–shell NPs are good candidates as positive CT imaging contrast agents. In addition, the high absorption coefficient of the AuNR@PAA/CaP yolk–shell NPs in the NIR region also makes them effective contrast agents for PA imaging. As shown in Fig. 5c, d, the PA signals of the NP solutions are enhanced with increasing NPs concentrations, displaying a concentration dependent manner. All the above results show that the AuNR@PAA/CaP yolk–shell NPs should be a promising candidate for dual-mode CT/PA imaging.

## Conclusions

In summary, we have successfully fabricated novel AuNR@PAA/CaP yolk–shell NPs which possess ultrahigh anticancer drug loading, superior photothermal conversion ability, good biocompatibility and pH/NIR dual-responsiveness. The yolk–shell NPs can serve as promising theranostic agents for simultaneous dual-mode CT/PA imaging and chemo-photothermal therapy. Furthermore, this work could encourage further study in the construction of CaP-based multifunctional yolk–shell NPs using NIR absorbing, fluorescent and magnetic nanomaterials for cancer theranostics.

## Electronic supplementary material

Below is the link to the electronic supplementary material.

Supplementary material 1 (PDF 717 kb)
