# Supplementary material for: Facile Approach to Synthesize Gold Nanorod@Polyacrylic Acid/Calcium Phosphate Yolk–Shell Nanoparticles for Dual-Mode Imaging and pH/NIR-Responsive Drug Delivery
Source: Nanomicro Lett. 2017 Oct 13;10(1):7. doi: 10.1007/s40820-017-0155-3 (PMC6199055; doi:10.1007/s40820-017-0155-3)
Supplement: Supplementary file 1 — Supplementary material 1 (PDF 717 kb) [file 40820_2017_155_MOESM1_ESM.pdf]

Supporting Information for

Facile Approach to Synthesize Gold Nanorod@Polyacrylic Acid/Calcium Phosphate Yolk-Shell Nanoparticles for Dual-Mode Imaging and pH/NIR-responsive Drug Delivery

Guilan Li[1], Yidan Chen[1], Lingyu Zhang[1], Manjie Zhang[1], Shengnan Li[1], Lu Li[1], Tingting Wang[2,] *, Chungang Wang[1,] *

[1]Faculty of Chemistry, Northeast Normal University, Changchun 130024, People's Republic of China
[2]School of Chemistry & Environmental Engineering, Changchun University of Science and Technology, Changchun 130022, People's Republic of China
*Corresponding authors. E-mail: wangcg925@nenu.edu.cn, wangtt@cust.edu.cn

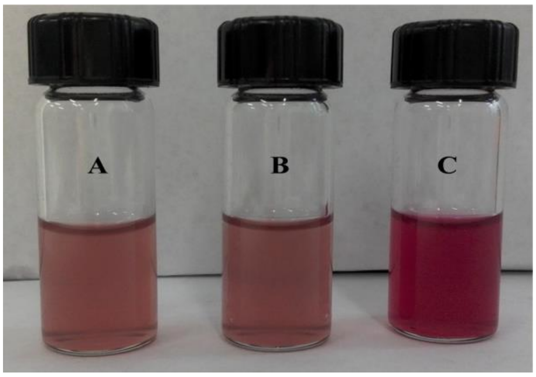

Fig. S1 Photographs of AuNR@PAA/CaP yolk-shell NPs in **A** water, **B** PBS buffer and **C** dulbecco's modified eagle medium (DMEM) and stored for 4 h, respectively

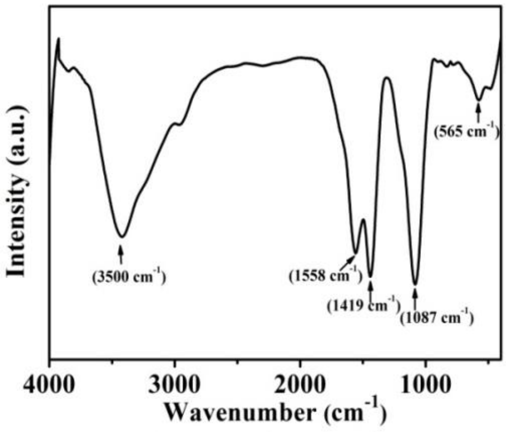

Fig. S2 FTIR spectrum of AuNR@PAA/CaP yolk-shell NPs

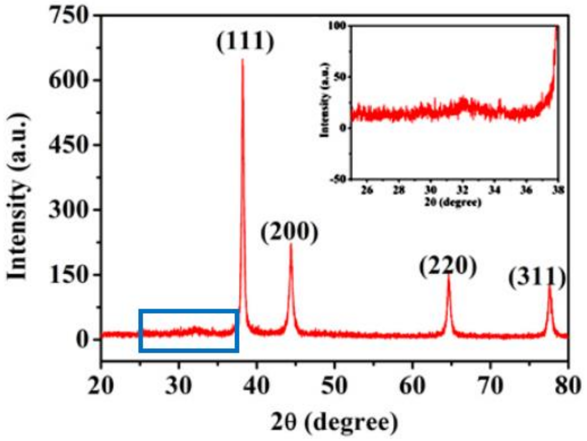

Fig. S3 XRD pattern of the AuNR@PAA/CaP yolk-shell NPs, the inset is the enlarged XRD pattern of the AuNR@PAA/CaP yolk-shell NPs.

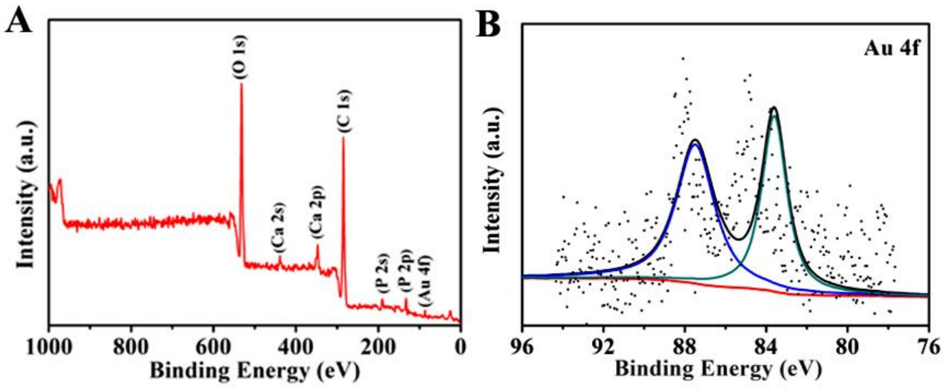

Fig. S4 XPS spectra of the AuNR@PAA/CaP yolk-shell NPs**: A** wide scan spectrum and **B** Au 4f

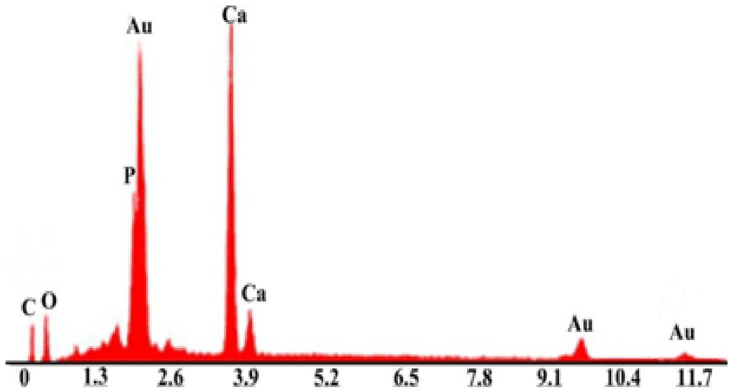

Fig. S5 EDX spectrum of AuNR@PAA/CaP yolk-shell NPs

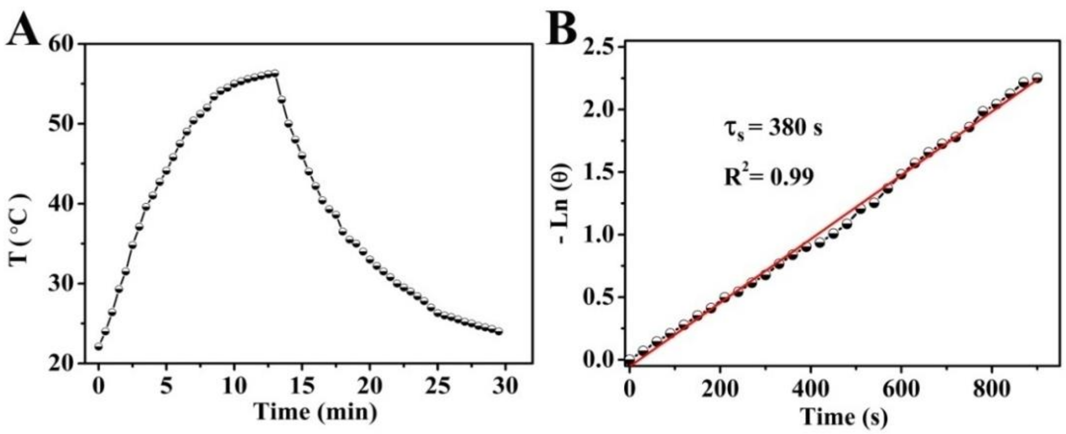

Fig. S6 A Photothermal effect of the irradiation of the aqueous dispersion of AuNR@PAA/CaP yolk-shell NPs (the Au concentration: 0.5 mM) with the NIR laser (808 nm, 1.0 W cm^{-2}), in which the irradiation lasted for 16 min, and then the laser was turned off. **B** Time constant for heat transfer from the system is determined to be τ_s = 380 s by applying the linear time data from the cooling period (after 16 min) versus negative natural logarithm of driving force temperature

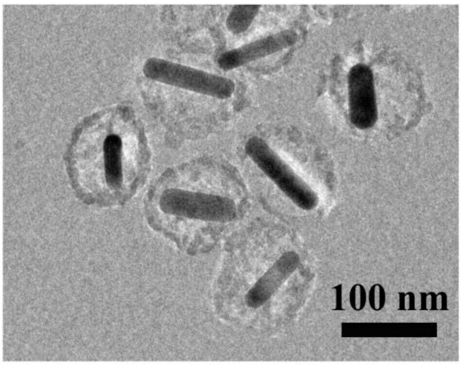

Fig. S7 The TEM image of AuNR@PAA/CaP yolk-shellNPs after four successive cycles of an on-and-off laser irradiation

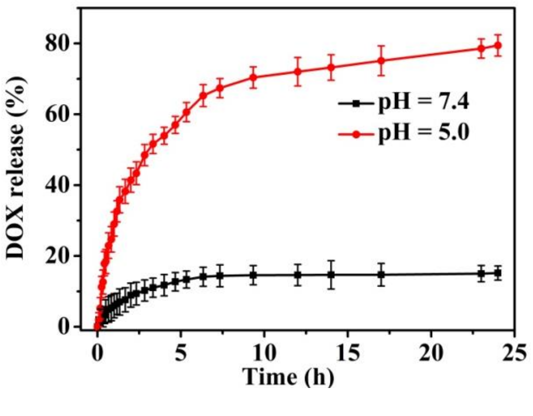

Fig. S8 DOX-release profiles of DOX from DOX-loaded AuNR@PAA/CaP yolk-shell NPs for 24 h in PBS buffer: pH 7.4 and pH 5.0

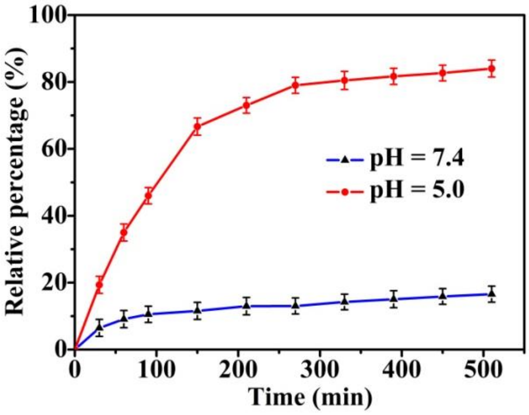

Fig. S9 The Ca content profiles for the AuNR@PAA/CaP yolk-shell NPs in the PBS (pH 5.0 and pH 7.4) at different time points at 37 ºC
